# Improving the quality of bowel preparation by smartphone education platform prior to colonoscopy: a randomized trail

**DOI:** 10.1080/07853890.2022.2130972

**Published:** 2022-10-18

**Authors:** Kai Zhao, Ruonan Dong, Suhong Xia, Lina Feng, Wangdong Zhou, Mingyu Zhang, Yu Zhang, Dean Tian, Mei Liu, Jiazhi Liao

**Affiliations:** Department of Gastroenterology, Tongji Hospital of Tongji Medical College, Huazhong University of Science and Technology, Wuhan, China

**Keywords:** Bowel preparation, colonoscopy, Boston bowel preparation score, education platform

## Abstract

**Objective:**

Adequate bowel preparation is an essential factor in colonoscopy. Enhanced education on the procedure of bowel preparation is very necessary for patients before colonoscopy. We analysed the influence of a novel education platform on bowel preparation quality before colonoscopy.

**Patients and Methods:**

The study enrolled outpatients who underwent colonoscopy in the digestive endoscopy centre of Wuhan Tongji Hospital. They were divided into the control group and the intervention group according to different educational ways. The control group patients were provided with the regular colonoscopy preparation leaflet. The intervention group patients were asked to add the education platform. The primary outcome was the rate of adequate bowel preparation. The study was registered at Chinese ClinicalTrials.gov (ChiCTR2100053547, 24/11/2021).

**Results:**

A total of 378 patients who underwent colonoscopy were enrolled, including 189 patients in the control group and 189 patients in the intervention group. The Boston bowel preparation score (BBPS) was significantly higher in the intervention group than that in the control group (*p* < .05). The adequate rate of bowel preparation in the intervention group was significantly improved than that in the control group (*p* = .000). Compared with the control group, the polyp detection rate (PDR) was significantly higher in the intervention group (*p* = .006), especially in the left colonic (*p* = .006). Among constipation patients, the adequate rate of bowel preparation (*p* = .000) and the PDR (*p* = .004) were significantly improved than that in the control group.

**Conclusions:**

The smartphone education platform may effectively improve bowel preparation quality, PDR, and patients’ compliance.

**Key messages**
- The quality of bowel preparation mainly relies on the patients’ compliance with the bowel preparation instructions.
- The study reveals that the superiority of the smartphone education platform by Mini Program in improving bowel preparation and colorectal polyp detection rate.
- The smartphone education platform may provide a more effective, convenient, and labour-saving way to provide further improvements to patients prior to colonoscopy.

## Introduction

Colonoscopy is considered as an important tool of screening, diagnosis, and treatment of colorectal lesions, such as adenoma, polyp, and early cancer, which has shown great potential to reduce the burden of advanced colorectal cancer [1]. Colonoscopy requires adequate bowel preparation, which contributes to the improvement of the identification of colorectal neoplasia and the decrease of the risk of missing lesions and post-colonoscopy colorectal cancer. Failed bowel preparation, on the other hand, could result in a lower likelihood of colorectal adenoma, a longer colonoscopy procedure time, a shorter interval between examinations, and a higher risk of colonoscopy-related adverse events [2,3]. Unfortunately, up to 20–25% of colonoscopies are reported to have failed bowel preparation [4].

The quality of bowel preparation mainly relies on the patients’ compliance with the bowel preparation instructions. Approximately 18–23.5% of patients are reported to fail to follow the bowel preparation instructions[5–7]. Enhanced education on the procedure of bowel preparation is very necessary for patients prior to colonoscopy. The guidelines suggest the use of enhanced instructions for bowel preparation[8,9]. At present, education leaflets on bowel preparation are regularly used in clinical practice. The instructions are too simple and most patients could not clearly understand the procedure of bowel preparation. At recent decades, several studies have proved that several methods could enhance patients’ compliance with instructions for bowel preparation, such as telephone, short message service (SMS), WeChat, application (APP), etc. [10–14]. Nevertheless, telephone and SMS require medical staff to spend more time and energy to instruct and supervise patients’ bowel preparation, which are not suitable for practical clinical work. WeChat official accounts platform mainly uploads relevant videos to instruct patients’ bowel preparation[15], which is limited in the interactive channel of communication between medical staff and patients. APP allows medical staff to have interactions with patients and provide feedback according to patients’ questions. It may not be conducive to the promotion of clinical use due to downloading the APP. Mini Program is a new open source of WeChat, and developers may quickly develop a new Mini Program. Mini Program may be easily accessed and disseminated in WeChat. Considering that WeChat is the most widely used social application in China, and Mini Program is similar to APP, which may be more beneficial for promotion and application. So, we use the Mini Program of WeChat to build a smartphone education platform to guide bowel preparation.

The study aimed to evaluate the impact of the smartphone education platform by Mini Program on guiding bowel preparation before colonoscopy.

## Patients and methods

### Study design and setting

This clinical trial was a single-blinded, randomized, and controlled trial, which was conducted by the Digestive Endoscopy Centre of Tongji Hospital in China. This study followed the principles of the Declaration of Helsinki and was approved by the Ethical Committees of Tongji Hospital of Tongji Medical College, Huazhong University of Science and Technology. Written informed consents were obtained from all of the patients (8/7/2021). The study was registered at Chinese ClinicalTrials.gov (ChiCTR2100053547, 24/11/2021).

### Patients

The study enrolled outpatients age ≥18 years who underwent colonoscopy in Tongji Hospital. Exclusion criteria: (1) history of colorectal surgery; (2) severe heart failure (New York Heart class II-IV); (3) chronic renal failure (stage 2-5) or mental disorder; (4) patients or his/her family members without a smartphone; (5) patients who did not provide written informed consent.

### Randomization and masking

At the time of appointment for colonoscopy, eligible patients were randomly distributed (1:1) into the control group or intervention group by using computer-generated random numbers, which were created by a data manager who was not involved in the colonoscopy procedure and data collection. In the control group, patients received traditional education way of bowel preparation (colonoscopy preparation brochure). In the intervention group, patients clicked the link to enter the education platform by Mini Program. Colonoscopies were done in the morning by experienced endoscopists who were unaware of the instructions that subjects received.

### Preparation of education interventions and preparation regimens

**Figure 1.**
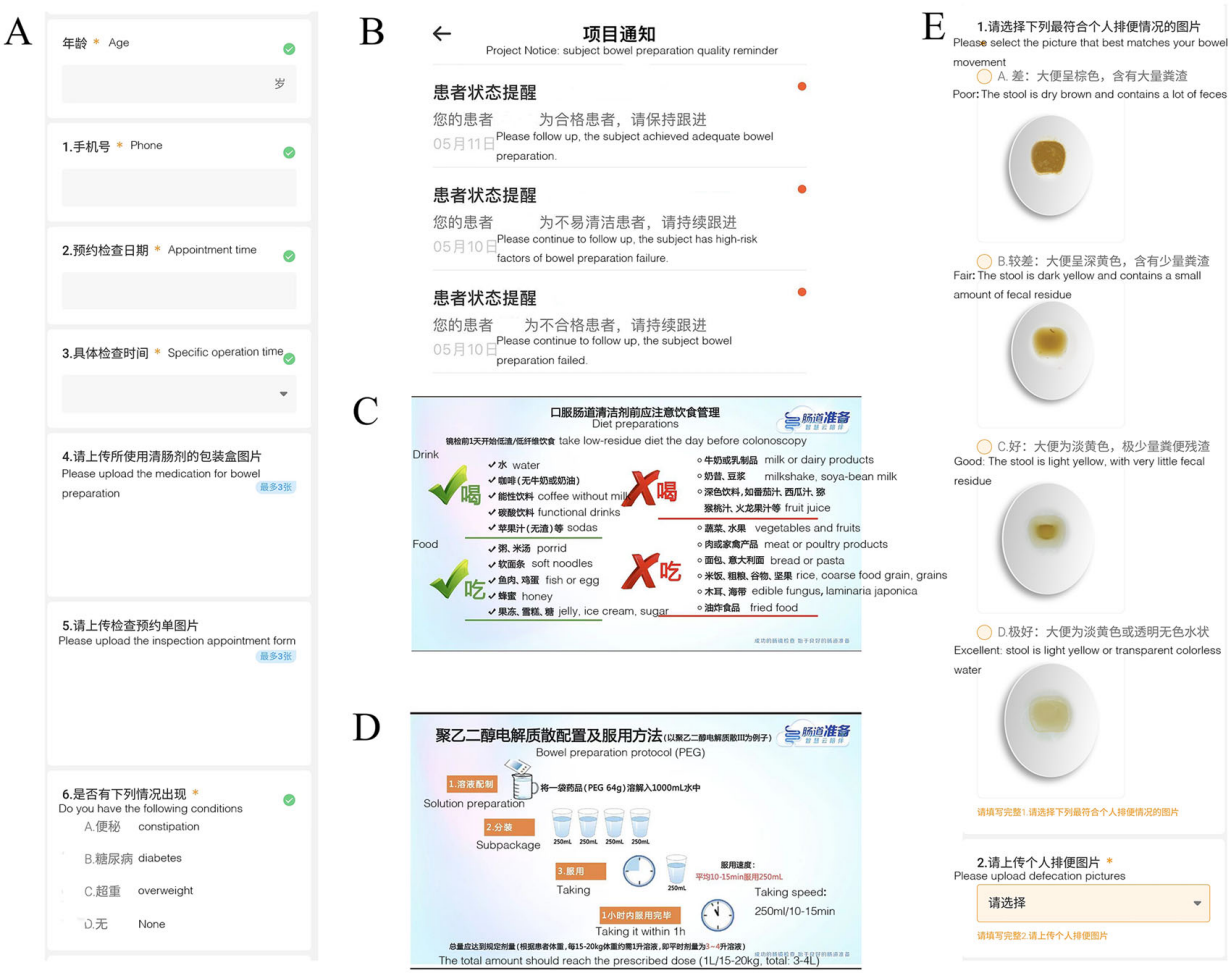
Education platform of mini program. (A) patient’s characteristics; (B) feedback the results; (C) dietary recommendations; (D) bowel preparation instruction; and (E) different levels of bowel preparation.

We used a novel smartphone education platform to guide bowel preparation. Patients assigned to the intervention group were instructed to add the education platform by Mini Program. First, each subject filled individual baseline characteristics (such as age, sex, laxatives, previous history) and operation time (Figure 1(A)). The platform could evaluate whether subjects had high-risk factors of bowel preparation failure (such as old age, obesity, diabetes, and constipation) according to the collected data, and then judged whether they are high-risk subjects of bowel preparation failure. The platform feedbacked the results to medical staff (Figure 1(B)). Subsequently, the subjects received links to information on bowel preparation, such as the importance of colonoscopy, operation process, dietary restrictions, bowel preparation schedule, criteria for successful bowel preparation, etc. (Figure 1(C,D)). The promotional materials were mainly presented in visual pictures and video materials, which were mainly developed by senior gastroenterologists and nurses according to the Chinese bowel preparation guidelines [9]. After completing bowel preparation, the subjects selected the results of bowel preparation (Figure 1(E)). The platform could assess whether the subjects needed to further strengthen bowel preparation and feedback to the subjects (Figure 1(B)). According to the feedback results, medical staff could further guide patients to perform bowel preparation to achieve qualified bowel preparation through online or telephone communication. Finally, the subjects’ satisfaction with the process of bowel preparation and the way of education were evaluated.

All patients received routine bowel preparation protocol (3 L PEG). At 10:00 pm before colonoscopy, two sachets of PEG were dissolved in 2 L warm water and were drunk within 1 h. At 5:00 am on the day of colonoscopy, another sachet was dissolved in 1 L warm water and was drunk within 30 min.

### Outcomes

The primary outcome was the quality of bowel preparation. During the colonoscopy, the quality of bowel preparation was assessed by using the Boston bowel preparation score (BBPS)[16]. The preparation of each colonic segment, divided into the right colon (cecum and ascending colon), the transverse colon, and the left colon (descending colon and rectosigmoid colon) were rated from 0 to 3. The worst cleanliness score was 0 points. The adequate bowel preparation was defined as BBPS ≥6 with each segment ≥2. The whole colon preparation quality was divided into 3 grades: excellent (total score 8–9), good (total score 6–7 and each segment score ≥2), failure (total score <6 or but one or more colon segment score <2). Secondary outcome measures included the polyp detection rate (PDR), patients’ compliance and acceptability, and adverse effect.

### Statistical analysis

We evaluated the bowel preparation quality in our endoscopic centre from 2021.02 to 2021.08, we found that the rate of adequate bowel preparation in patients was about 70%. Based on assumptions of α = 0.05 and β = 0.1, and lost to a follow-up rate of 10%, we calculated that at least 189 patients in each group were needed to detect a statistically significant difference between group A and group B with a two-tailed. Achieving a 1:1 ratio in 2 groups, we estimated that a total of 378 patients would be adequate to detect a significant difference in the primary endpoint.

SPSS 26.0 was used for the statistical analysis (SPSS, Chicago, IL). Continuous variables were expressed as means with standard deviation (SD) and analysed using one-way ANOVA, and t-test. Categorical variables were analysed using the person chi-square test. A *p*-value <0.05 was considered statistically significant.

## Results

### Baseline characteristic

**Figure 2.**
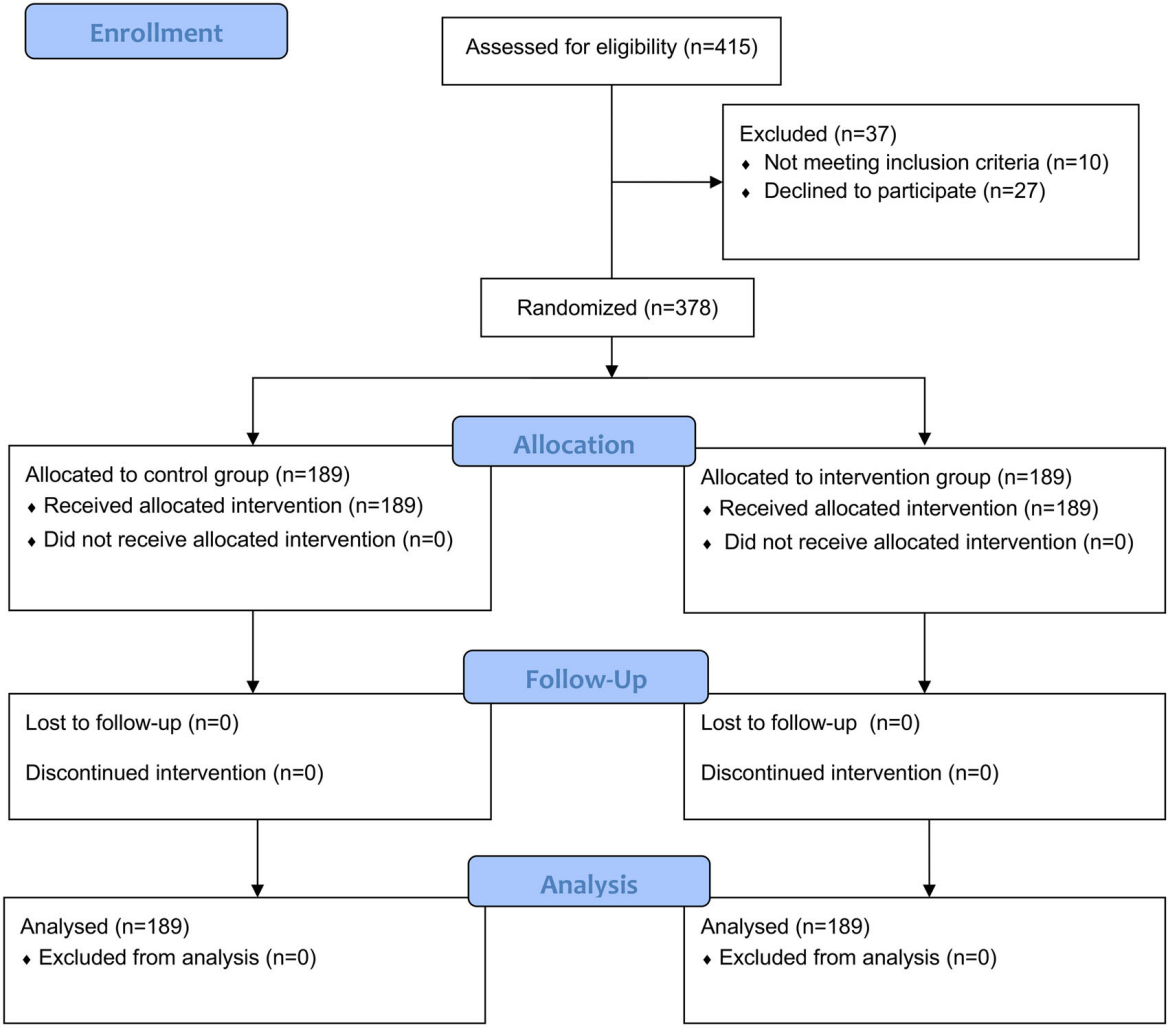
Flowchart of the study.

A total of 415 outpatients were assessed for underwent colonoscopy during the study period. After exclusion of 37 individuals who declined to participate or who fulfilled the exclusion criteria, 378 patients who underwent colonoscopy were enrolled, and allocated according to the randomization number table in a 1:1 ratio (*n* = 189) (Figure 2). All outpatients underwent colonoscopy by experienced endoscopists in the morning. The baseline features of all patients were shown in Table 1. We found that there were no significant differences among the two groups upon the baseline characteristics except for age (*p* > .05). The subjects in the intervention group were younger (*p* = .000).

**Table 1.** Baseline characteristics of the study patients.

	Control group n (%)	Intervention group *n* (%)	*p* Value
Age, years, mean (SD)	49.6 ± 12.3	43.4 ± 11.2	.000
Sex (Male)	89 (47.1%)	96 (50.8%)	.471
BMI	22.6 ± 3.0	22.6 ± 2.7	.761
Smoking	38 (20.1%)	37 (19.6%)	.897
Drinking	25 (13.2%)	20 (10.6%)	.427
Indication for colonoscopy			.693
Constipation	63 (33.3%)	55(29.1%)	
Abdominal discomfort	72 (38.1%)	70 (37.0%)	
Screening	32 (16.9%)	39 (20.6%)	
Others	22 (11.6%)	25 (13.2%)	

Values are given as mean ± SD or *n* (%).

### Quality of bowel preparation

Table 2 shows the quality of bowel preparation. Compared with the control group, the intervention group obtained significantly higher total BBPS scores (7.5 ± 1.4 vs 6.2 ± 1.5, *p* = .000). The intervention group showed significantly higher BBPS score at each colon segment than the control group (*p* < .05). The adequate preparation rates in the right and left colon were significantly higher in intervention group than that in the control group (92.1% vs 80.4, *P*_left_=0.001; 92.6% vs 81.5%, *P*_right_=0.001). In the transverse colon, there was no significant difference between the two groups (95.8% vs 91.0%, *p* = .063). The rate of excellent and good bowel preparation in the intervention group was higher than that in the control group (92.1% vs 79.9%, *p* = .000).

**Table 2.** Bowel preparation quality.

	Control group *n* (%)	Intervention group *n* (%)	*p* Value
BBPS score			
Left colon	1.8 ± 0.5	2.5 ± 0.6	.000
Transversum colon	2.4 ± 0.7	2.6 ± 0.6	.001
Right colon	2.0 ± 0.6	2.4 ± 0.7	.000
Total score	6.2 ± 1.5	7.5 ± 1.4	.000
Adequate bowel preparation rate			
Left colon	152 (80.4%)	174 (92.1%)	.001
Transversum colon	172 (91.0%)	181 (95.8%)	.063
Right colon	154 (81.5%)	175 (92.6%)	.001
Quality of bowel preparation			.000
Excellent	27 (14.3%)	104 (55.0%)	
Good	124 (65.6%)	70 (37.0%)	
Failure	38 (20.1%)	15 (7.9%)	

Values are given as mean ± SD or *n* (%).

BBPS: Boston bowel preparation score.

### Outcome of colonoscopy

The outcomes of colonoscopy are shown in Table 3. In the intervention group, the successful cecal intubation rate was 94.2%, which was significantly higher than that in the control group (87.3%, *p* = .021). The colorectal polyp detection rate (PDR) in the intervention group was significantly higher than that in the control group (39.7% vs 26.5%, *p* = .006). Regarding the segmental colon, the PDR of the left colon was significantly higher in the intervention group than that in the control group (33.9% vs 21.2%, *p* = .006).

**Table 3.** Outcomes of colonoscopy.

	Control group *n* (%)	Intervention group *n* (%)	*p* Value
Caecal intubation rate	165 (87.3%)	178 (94.2%)	.021
PDR	50 (26.5%)	75 (39.7%)	.006
Location of polyp			
Left colon	40 (21.2%)	64 (33.9%)	.006
Transversum colon	12 (6.9%)	22 (11.1%)	.150
Right colon	14 (7.4%)	19 (10.1%)	.362
Other lesions			
IBD	11 (5.8%)	9 (4.8%)	
Colitis	3 (1.6%)	10 (5.3%)	
Diverticula	1 (0.5%)	5 (2.6%)	

Values are given as mean ± SD or *n* (%).

PDR: polyp detection rate;IBD: inflammatory bowel disease.

### The subgroup analysis for the control group and the intervention group

We further analysed the quality of bowel preparation for constipation patients (Table 4). In the constipation subgroup, the total BBPS score of the intervention group was significantly higher than that in the control group (7.3 ± 1.8 vs 5.9 ± 1.8, *p* = .000). The PDR was significantly higher in the intervention group than that in the control group (39.3% vs 15.9%, *p* = .004), and the PDR of the left colon in the intervention group was also improved (30.4% vs 12.7%, *p* = .018).

**Table 4.** The subgroup of constipation patients.

	Control group *n* (%)	Intervention group *n* (%)	*p* Value
BBPS score			
Left colon	1.7 ± 0.5	2.4 ± 0.6	.000
Transversum colon	2.3 ± 0.7	2.6 ± 0.7	.022
Right colon	1.9 ± 0.7	2.3 ± 0.8	.002
Total score	5.9 ± 1.8	7.3 ± 1.8	.000
Adequate bowel preparation			
Left colon	43 (68.2%)	50 (89.3%)	.006
Transversum colon	53 (84.1 %)	52 (92.9%)	.140
Right colon	46 (73.0%)	51 (91.1%)	.011
Quality of bowel preparation			.000
Excellent	9 (14.3%)	30 (53.6%)	
Good	34 (54.0%)	20 (35.7%)	
Failure	20 (31.7%)	6 (10.7%)	
PDR	10 (15.9%)	22 (39.3%)	.004
Location of polyp			
Left colon	8 (12.7%)	17 (30.4%)	.018
Transversum colon	4 (6.3%)	7 (12.5%)	.248
Right colon	2 (3.2%)	6 (10.7%)	.101

Values are given as mean ± SD or *n* (%).

BBPS: Boston bowel preparation score; PDR: polyp detection rate.

### Patients’ adverse effects, compliance, and acceptability

The adverse effects, compliance, and acceptability are presented in Table 5. There was no significant difference in the incidence of adverse effects between the two groups (*p* = .631). The evaluation score of instruction was lower in the control group than that in the intervention group (3.3 ± 1.0 vs 4.0 ± 1.0, *p* = .000). Patients were more aware of the process of bowel preparation in the intervention group (94.7% vs 84.1%, *p* = .010). The rate of willingness to repeat the same instruction of bowel preparation was significantly higher in the intervention group than that in the control group (*p* = .010). Compared with the control group, compliance was better in the intervention group (*p* = .042).

**Table 5.** Patients’ adverse effect, acceptability and compliance.

	Control group *n* (%)	Intervention group *n* (%)	*p* Value
Adverse effect			.631
Nausea and vomiting	19 (10.1%)	12 (6.3%)	
Abdominal distension	3 (1.6%)	3 (1.6%)	
Abdominal pain	2 (1.1%)	3 (1.6%)	
Acceptability			
Evaluation (0–5 scale)	3.3 ± 1.0	4.0 ± 1.0	.000
Willingness to repeat	159 (84.1%)	175 (92.6%)	.010
Clearness	163 (86.7%)	179 (94.7%)	.007
Compliance	171 (90.5%)	181 (95.8%)	.042

Values are given as mean ± SD or *n* (%).

## Discussion

Colonoscopy is considered as a gold standard of screening, diagnosis, and treatment of colorectal lesions[1]. As the use of colonoscopy for colon diseases is spreading, an increasing amount of attention has been paid to the quality of this examination. Multi-step bowel preparation and patients’ poor compliance are the risk factors for inadequate bowel preparation quality[5–7]. Some reinforced education methods have been developed to improve patient’s compliance to increase the quality of bowel preparation. Previous studies showed that Application for reinforced education of bowel preparation was feasible and could lead to high BBPS scores and patient satisfaction[13,14,17,18]. Our study also showed that the education platform based on the smartphone may significantly improve the quality of bowel preparation, PDR, and patients’ compliance. The results showed that the mode of information delivery and interchange are important variables in effectively gaining information and carrying it out.

With the rapid development and popularity of smartphones in China, smartphone-related health information sites and applications were developed and promoted, such as health knowledge popularization, health consultation, outpatient registration, etc. Compared with the previous methods, such as telephone, SMS, and Application, the smartphone multi-functional education platform by Mini Program of WeChat was easy to implement and could reduce the workload of medical staff, which was more adapt to the specific needs of current medical resources shortage in China.

The more effective outcomes in the intervention group may be due to the delivery of more comprehensive content conveyed by educational videos and physician-patient communication compared to education brochures. We found that the rate of adequate bowel preparation in the intervention group was superior than that in the control group (*p* < .05). Patients receiving new education instruction based on the smartphone had higher PDR compared with patients using an educational brochure. Considering the location of polyp, we found that the left colon PDR of patients using new education instruction were higher than patients using an educational brochure (*p* < .0.5). Alvarez-Gonzalez *et al.* [19] reinforced education by telephone within 48 h before the colonoscopy among patients with previous inadequate bowel preparation, they found that the quality of bowel preparation in the left colon was improved than that in other colon segments, and the PDR of the left colon was significantly higher in the intervention group. This might be due to inadequate bowel preparation and larger sacculation in the left colon. In our study, we reveal that the superiority of the smartphone education platform in improving the bowel preparation of the left colon may compensate for the disadvantage of its anatomical structure. However, Water *et al.* [20]. found that the quality of bowel preparation and the PDR in the right colon were substantially improved among patients with strengthened patients’ education by using SMS. However, we choose 3 L rather than low-volume PEG in our study, following the evidence from Chinese guidelines for bowel preparation for colonoscopy. The bowel preparation capacity was higher than that in Water’s study, which caused an impact on the research results.

The risk factors of inadequate bowel preparation include elder patients, chronic constipation, BMI> 25 kg/m,^2^ colorectal surgery history, Parkinson’s diseases, diabetes, etc. [21]. The education platform could identify whether patients were with the risk factors and provide feedback to the medical staff. According to the feedback results, medical staff may focus on guiding the high-risk population with bowel preparation failure. We further analysed the quality of bowel preparation of patients with constipation, we found that adequate bowel preparation and PDR were significantly improved in the intervention group than those in the control group. The result showed that strengthened bowel preparation education could result in a significant improvement in bowel preparation among patients with risk factors of inadequate bowel preparation. Alvarez-Gonzalez *et al.* [19] study showed that bowel preparation was significantly improved among patients with previous inadequate bowel preparation. There, enhanced education on the procedure for bowel preparation could be considered as a beneficial strategy for the high-risk population with bowel preparation failure. However, we found that the subjects in the intervention group were younger (*p* < .05), the reason was that there were fewer older subjects in the intervention group (28.6% vs 54.0%). Previous research showed that people older than 60 used the telephone (74.2%) more frequently than SMS (35.8%)[22]. Therefore, it is necessary for us to further optimize the Mini Program in the future.

In our study, we found that the Mini Program of WeChat could significantly improve bowel preparation quality, PDR, and patients’ compliance. At the same time, patients are more clearing about the bowel preparation process and willing to repeat the same instruction. The education platform of Mini Program could not only guide patients to achieve high-quality bowel preparation through text, education videos, and medical staff patients’ communication but also identified high-risk patients with failed bowel preparation and feedback to medical staff, which play the value of medical staffs to provide medical guidance to patients outside the hospital. The Mini Program, as a way of auxiliary medical education, plays a positive role in both strengthening medical guidance and provides a way for doctors to communicate with patients outside of hospital. Therefore, this method is worth opening and promoting in the preliminary preparation of most medical behaviours.

Our study also had some limitations. First of all, the study was a single-centre study, which limited its generalisability. We are interested in conducting further research at multi-centres and large sample size levels to evaluate the effectiveness of the education method. Secondly, although smartphones are pervadingly used in society, we found that there were some elderly patients who have certain difficulties for using smartphones. Therefore, it is necessary to develop an optimal method to improve bowel preparation for elder patients. Lastly, we will compare our education platform of the smartphone with other education methods, such as telephone, SMS, WeChat, etc. to evaluate the superiority in the future.

## Conclusions

In summary, our study reveals that the superiority of the smartphone education platform by Mini Program in improving bowel preparation and colorectal polyp detection rate, especially the proximal colon polyps. Therefore, the smartphone education platform may provide a more effective, convenient, and labour-saving way to provide further improvements for patients prior to colonoscopy.

## Data Availability

The data analysed for this study can be accessible from the corresponding author on reasonable request.

## Appendices

CONSORT 2010 checklist of information to include when reporting a randomized trial*
